# Mediating effects of lipids on the association between smoking and coronary artery disease risk among Chinese

**DOI:** 10.1186/s12944-020-01325-4

**Published:** 2020-06-23

**Authors:** Wenjing Song, Jieqiong Guan, Pan He, Siyu Fan, Hong Zhi, Lina Wang

**Affiliations:** 1 Key Laboratory of Environmental Medicine Engineering, Ministry of Education, Department of Epidemiology & Biostatistics, School of Public Health, Southeast University, 87 Ding Jiaqiao Rd, Nanjing, 210009 China; 2 Department of Cardiology, ZhongDa Hospital, Southeast University, Nanjing, 210009 China

**Keywords:** Smoking, Lipid, Coronary artery disease, Mediation analysis, Casual inference

## Abstract

**Objective:**

The mechanism between smoking and coronary artery disease (CAD) remains unclear. It is likely that lipid (including triglycerides (TG), total cholesterol (TC), low density lipoprotein-cholesterol (LDL-C), high density lipoprotein-cholesterol (HDL-C)) have been functioning as one of the mediators between smoking and the CAD occurrence. The study aimed to investigate the mediating effect of lipid on the relationship between smoking and CAD risk.

**Methods:**

The case-control study included 2048 subjects. General linear regression analysis was used to corroborate the association between smoking and lipid levels. Univariate and multivariate logistic regression analysis were performed to reveal the relationship between smoking, lipid and the risk of CAD. Mediation analysis was used to investigate whether the association between smoking and CAD risk was mediated by lipid.

**Results:**

Smoking was found to be associated with the risk of CAD (odds ratio (OR) = 1.34, 95% confidence interval (CI): 1.05–1.71, *P* = 0.019). Regression analysis showed that TG, TC and HDL-C were associated with CAD (OR = 2.69, 95%CI: 2.12–3.40, *P* < 0.001; OR = 0.34, 95%CI: 0.29–0.43, *P* < 0.001; OR = 0.37, 95%CI: 0.30–0.47, *P* < 0.001). Moreover, the ratio of TG to HDL-C (TG/HDL-C) was also related to CAD (OR = 4.45, 95%CI: 3.52–5.64, *P* < 0.001). Mediation analysis showed that among the effects of smoking on CAD, 17.52% was mediated by lipid, in which HDL-C accounted for 11.16% and TG accounted for 6.36%. Further analysis showed that the effect was also partially mediated by TG/HDL-C, which was accounted for 28%.

**Conclusions:**

Lipid plays a partial mediation on the association between smoking and CAD risk. The study provides a clue on the mediation effect of lipids on the relationship between smoking and CAD risks, which is a novel insight to the progression of CAD.

## Introduction

Cardiovascular diseases (CVDs) are the major cause of death in the world and the main obstacle of human sustainable development [1–3]. CAD is the most important component of CVDs, which is considered to be a leading public health burden [4, 5]. It is estimated that in the year 2018, about 720,000 individuals in the United States experienced new coronary events [6]. It was reported that 11 million Chinese suffer from CAD, and the incidence and mortality of CAD are still raising [7]. Therefore, it is very important to explore the influencing factors and pathogenesis of CAD for improve people’s quality of life.

Traditional epidemiological studies have shown that dyslipidemia and smoking are associated with the development of CAD [8, 9]. The pathological basis of CAD is atherosclerosis and lipoproteins play critical roles in the process of atherosclerosis [10]. Therefore, an increased level of LDL-C, TC, TG and a decreased level of HDL-C are associated with an increased risk of CAD [11]. A retrospective cohort study found that TG/HDL-C contributed significantly to the prediction of long-term mortality in patients with CAD after undergoing percutaneous coronary intervention [12]. Cigarette smoking causes about 6 million deaths worldwide every year, and CVD accounts for 40% of all the smoking-related deaths [13]. Cigarettes are a mixture of several toxic chemicals, such as nicotine, carbon monoxide and so on. Heavy smoking is associated with an increased risk of CAD [14]. However, the mechanism by which smoking causes CAD remains unclear. Some studies suggest that lipids might play an important role in the pathway from smoking to CAD progression [6], even if the hypothesis has not been fully proven.

In the field of psychology and other social sciences, a large number of papers analyze the mechanism of independent variables on dependent variables by establish mediating models. Mediation analysis has also been recently applied in the field of epidemiology. Fan Wang et al. [15] found that the association between APOA5 variant and CAD was partly mediated by TG and HDL-C through mediation analysis. Moreover, Tao Wang et al. [16] found that white fibrinogen, blood cells and hs-CRP partially mediate the association between smoking and intima-media thickness by mediation analysis.

Therefore, Mediation analysis was used to explore the association between smoking and CAD. Further analysis to determine whether lipid is a mediator on the association between smoking and CAD among Chinese.

## Methods

### Ethics statement

All participants provided informed consent. This study was approved by the Ethics Committee of Clinic Medical College, Southeast University, Nanjing, China (Project identification code: 81673259, BK20161435).

### Study population

The present study included 1024 CAD patients (cases group) and 1024 non-CAD subjects (control group). CAD group were recruited between September 2011 to December 2018 in ZhongDa Hospital Affiliated to Southeast University, Nanjing, China. Non-CAD group were selected from the healthy people who underwent physical examination from March to April 2019 in Qixia district, Nanjing, China. CAD subjects received quantitative coronary angiography with a cardiovascular measurement system. CAD patients were defined as those having angiographic coronary stenosis of at least 50% lumen reduction in at least one major epicardial coronary artery.

Information about demographic data, lifestyle and the vascular events history were obtained using a structured questionnaire. Clinical history from each subject, including sex, age, body mass index (BMI), co-morbidities (Hypertension(HBP), Diabetes mellitus(DM)), white blood cells (WBC), bilirubin, smoking and alcohol drinking were collected. Smokers were defined as individuals who smoked at least one cigarette per day for 1 year or more than 1 year, and had not quit before they participated in the study.

### Lipid levels detection

Venous blood samples were taken after after 12–14 h of fasting. The samples were collected in 0.1% EDTA-containing tubes for lipoprotein measurements. The concentrations of TC, HDL-C, LDL-C and TG were determined by standard biochemical methods using a chemistry analyzer (Synchron clinical system LX20, Beckman Coulter Inc., California. USA).

### Statistical analysis

Continuous variables were presented as mean and standard deviation (SD). Categorical variables were expressed by counts and percentages (%). The one-way analysis of variance and Pearson’s χ2 test were conducted to compare the participants’ baseline characteristics. The relationship between smoking and lipid was corroborated by general linear regression analysis. To assess the association between lipid, smoking and the risk of CAD, univariate and multivariate logistic regression analysis were performed. In the logistic regression analysis, the continuous variables were divided into the low-level group and the high-level group according to the median of the non-CAD group. Statistical significance was defined as *P* < 0.05 (two sides). Analyses were carried out with the SPSS program (V 23.0, SPSS, IBM, Illinois. USA).

To determine whether lipid could mediate the effect of smoking on the CAD risk, the PROCESS procedure in SPSS was used for mediation analysis, where the mediator should be the continuous variable [17]. The mediation analysis model can be represented by the following three regression equations. Equation (1) shows the total effect of X on Y, Eq. (2) explains the effect of X on M, Eq. (3) reveals the relationship between X and Y adjusted for M and the association between M and Y adjusted for X. e_1_, e_2_, and e_3_ are residuals for the respective equations [18]. The mediation analysis in this study was divided into two models (modle1: the mediators were TG and HDL-C; modle2: the mediator was TG/HDL-C).

1 $$ \mathrm{Y}=c\mathrm{X}+{\mathrm{e}}_1 $$

2 $$ \mathrm{M}=a\mathrm{X}+{\mathrm{e}}_2 $$

3 $$ \mathrm{Y}=c^{\prime}\mathrm{X}+b\mathrm{M}+{\mathrm{e}}_3 $$

## Results

### Characteristics of study participants

1024 CAD and 1024 non-CAD participants were 1:1 matched by age (±3 years) and gender. The clinical characteristics of the selected subjects are provided in Table 1. There was no significant difference of the age between the cases and controls (*P* = 0.303). 32.62% of the cases and 25.78% of the normal controls were smokers. Compared with the control group, the average level of TG among the cases was significantly higher (*P* < 0.001), while the average level of HDL-C among cases were lower than control group (*P* < 0.001), so TG/HDL-C was a little bit higher among CAD patients (*P* < 0.001). However, the TC levels in the cases group were relatively lower than those in the control group. In addition, borderline difference of the LDL-C between cases and controls was found (*P* = 0.086). Among the CAD patients, 59.28 and 84.38% cases had been treated with statins and antiplatelet drugs, respectively.

**Table 1:** Baseline characteristics of CAD patients and Non-CAD

		Total (*N* = 2048, %)	CAD (*N* = 1024, %)	Non-CAD (N = 1024, %)	*P*
Gender	Male	1024(50)	512(50)	512(50)	1.000
	Female	1024(50)	512(50)	512(50)	
Age	Mean ± SD	64.21 ± 7.33	64.38 ± 7.45	64.04 ± 7.19	0.303
BMI (kg/m^2^)	Mean ± SD	23.90 ± 2.73	24.21 ± 2.66	23.59 ± 2.77	< 0.001
Smoking	Yes	597(29.20)	334(32.62)	263(25.68)	< 0.001
	No	1451(70.80)	690(67.38)	761(74.32)	
Drinking	Yes	277(13.50)	914(89.26)	857(83.69)	< 0.001
	No	771(86.50)	110(10.74)	167(16.31)	
HBP	Yes	1194(58.30)	695(67.87)	499(48.73)	< 0.001
	No	854(41.70)	329(32.13)	525(51.27)	
DM	Yes	434(21.20)	269(26.27)	165(16.11)	< 0.001
	No	1614(78.80)	755(73.73)	859(83.89)	
WBC (10^9^/L)	Mean ± SD	6.26 ± 1.71	6.52 ± 1.91	6.01 ± 1.43	< 0.001
Bilirubin(mg/L)	Mean ± SD	13.79 ± 5.76	13.14 ± 4.99	14.30 ± 5.39	< 0.001
TG (mmol/L)	Mean ± SD	1.44 ± 0.94	1.65 ± 1.01	1.22 ± 0.81	< 0.001
TC (mmol/L)	Mean ± SD	4.83 ± 1.10	4.52 ± 1.12	5.14 ± 0.98	< 0.001
LDL-C(mmol/L)	Mean ± SD	2.68 ± 0.77	2.71 ± 0.87	2.65 ± 0.65	0.086
HDL-C(mmol/L)	Mean ± SD	1.31 ± 0.38	1.21 ± 0.39	1.42 ± 0.34	< 0.001
TG/HDL-C	Mean ± SD	1.22 ± 0.98	1.47 ± 0.99	0.98 ± 0.91	< 0.001

Results are presented as mean ± SD or n (%)

*BMI* body mass index; *HBP* hypertension; *DM* diabetes mellitus; *TG* triglyceride; *TC* total cholesterol; *LDL-C* low density lipoprotein-cholesterol; *HDL-C* high density lipoprotein cholesterol; *WBC* white blood cells

**Table 2:** Multivariate regression analysis on the relationships between smoking, lipid and CAD risk

Variable	Model 1			Model 2		
	β	OR (95%CI)	*P*	*β*	OR (95%CI)	*P*
BMI (kg/m^2^)	−0.023	0.98(0.79–1.22)	0.836	0.013	1.00(0.80–1.24)	0.977
Smoking, Y/N	0.291	1.34(1.05–1.71)	0.019	0.384	1.38(1.08–1.75)	0.01
Drinking, Y/N	−0.705	0.49 (0.36–0.68)	< 0.001	− 0.709	0.48(0.35–0.66)	< 0.001
HBP, Y/N	0.563	1.76(1.43–2.17)	< 0.001	0.556	1.73(1.40–2.12)	< 0.001
DM, Y/N	0.300	1.35 (1.05–1.73)	0.018	0.35	1.37(1.07–1.76)	0.013
WBC (10^9^/L), H/L	0.220	1.25(1.01–1.53)	0.039	0.166	1.24(1.01–1.52)	0.044
Bilirubin(mg/L), H/L	−1.046	0.35(0.29–0.43)	< 0.001	− 0.94	0.49(0.40–0.61)	< 0.001
TG (mmol/L), H/L	0.989	2.69(2.12–3.40)	< 0.001	*	*	*
TC (mmol/L), H/L	−1.037	0.34(0.28–0.44)	< 0.001	− 0.253	0.36(0.29–0.44)	< 0.001
HDL-C (mmol/L), H/L	−0.989	0.37(0.30–0.47)	< 0.001	*	*	*
TG/HDL-C, H/L	*	*	*	1.415	4.45(3.52–5.64)	< 0.001

multivariate regression analysis was performed. The dependent variable was CAD. Modle1: the mediators were TG and HDL-C; Modle2: the mediator was TG/HDL-C. Variables showing significance value< 0.05 in univariate analysis were included in the multivariate analysis. *BMI* body mass index; *HBP* hypertension; *DM* diabetes mellitus; *TG* triglyceride; *TC* total cholesterol; *HDL-C* high density lipoprotein cholesterol; *WBC* white blood cells

### Association between smoking, lipids and CAD

Significant association were observed between smoking, TG, TC, HDL-C, TG/HDL-C and CAD after adjusting for the potential confounders (TG: OR = 2.69, 95% CI: 2.12–3.40, *P* < 0.001; TC: OR = 0.34, 95%CI: 0.28–0.44, *P* < 0.001; HDL-C: OR = 0.37, 95%CI: 0.30–0.47, *P* < 0.001; TG/HDL-C: OR = 4.45, 95%CI: 3.52–5.64, *P* < 0.001). Compared with the subjects in the lower levels of TG and TG/ HDL-C, the subjects in the high levels were associated with 1.69 times and 3.45 times increased risk of CAD, respectively. On the other hand, compared with the subjects with lower levels of TC and HDL-C, the risk of CAD in subjects with high levels decreased by 66 and 63%, respectively. No association was observed between the increased level of LDL-C and CAD risk (Table 2).

### Association between smoking and lipid levels

CAD was found to be positively correlated with TG (β = 4.845, *P* < 0.001) and TG/HDL-C (β = 0.121, *P* = 0.024), while it was negatively correlated with HDL-C (β = − 0.159, *P* < 0.001) (Table 3).

**Table 3:** General linear regression on the relationships between smoking and lipid levels

Variable	TG		TC		LDL-C		HDL-C		TG/HDL-C	
	β	*P*	β	*P*	β	*P*	β	*P*	β	*P*
Gender, M/F	0.015	0.586	0.523	< 0.001	0.276	< 0.001	0.169	0.058	−0.175	< 0.001
Age	0.001	0.746	0.003	0.411	0.002	0.316	−0.003	0.218	0.001	0.721
BMI (kg/m^2^)	0.098	< 0.001	0.019	0.041	0.017	0.008	−0.021	< 0.001	0.100	< 0.001
Smoking, Y/N	0.123	0.016	0.029	0.647	0.115	0.010	−0.046	0.025	0.121	0.024
Drinking, Y/N	−0.013	0.837	0.243	0.002	−0.018	0.737	0.100	< 0.001	−0.107	0.101
HBP, Y/N	0.156	< 0.001	−0.090	0.075	0.028	0.432	−0.025	0.124	0.137	0.001
DM, Y/N	0.205	< 0.001	−0.197	0.001	−0.100	0.017	−0.065	0.001	0.247	< 0.001
WBC	0.064	< 0.001	0.026	0.075	0.042	< 0.001	−0.024	< 0.001	0.066	< 0.001
Bilirubin(mg/L)	−0.006	0.086	0.002	0.654	−0.007	0.045	0.003	0.009	−0.008	0.032

General linear regression was performed. The dependent variable was lipid levels. *M*male; *F*female; *BMI* body mass index; *HBP* hypertension; *DM* diabetes mellitus; *TG* triglyceride; *TC* total cholesterol; *HDL-C* high density lipoprotein cholesterol; *WBC* white blood cells

### Mediating effect of lipid on the association between smoking and CAD

**Fig. 1:**
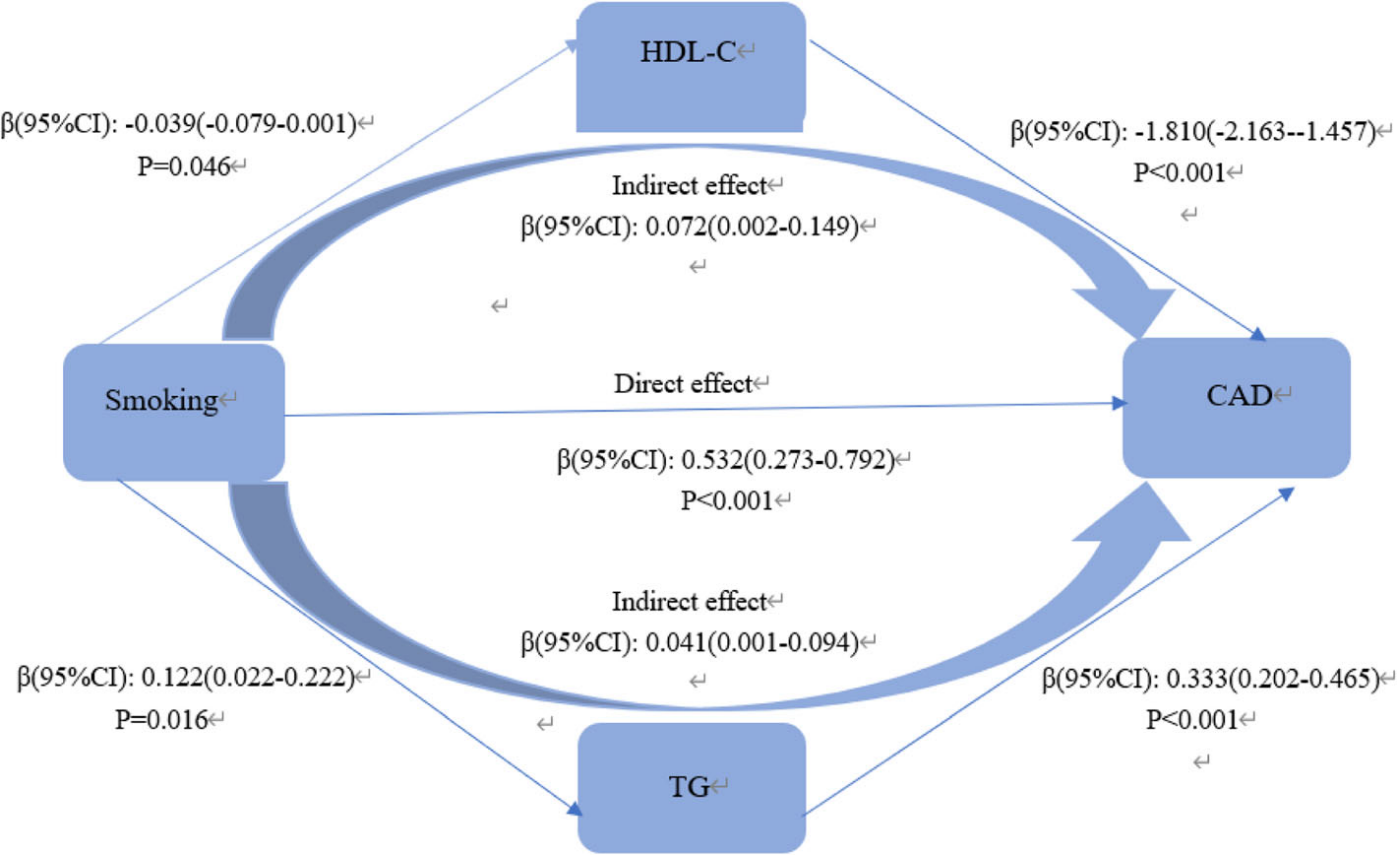
Mediating effect of TG, HDL-C on the relationship between smoking and CAD

**Fig. 2:**
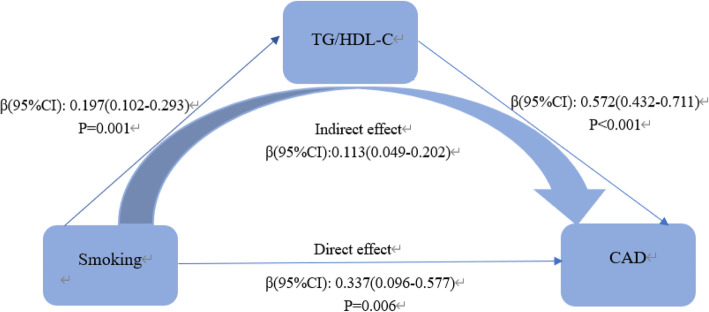
Mediating effect of TG/HDL-C on the relationship between smoking and CAD

Mediating analysis revealed the mediation effect of lipid on the relationship between smoking and CAD. HDL-C, TG, TG/HDL-C were considered as the mediators separately. In model 1, The total direct effect of smoking on CAD was significant (β = 0.532, 95%CI: 0.273–0.982). The association between cigarette smoking and CAD was partly mediated by HDL-C (β = 0.072, 95%CI: 0.002–0.149) and TG (β = 0.040, 95%CI: 001–0.094). So that, HDL-C and TG accounted for 11.16 and 6.36% on the correlation between smoking and CAD respectively (Fig.1). In addition, TG/HDL-C also partially mediated the effect of smoking on the increased risks of CAD, accounting for a mediation ratio of 28%. The coefficients of direct effect and indirect effect were 0.337(95%CI: 0.096–0.577) and 0.113(95%CI: 0.049–0.202) respectively (Fig.2).

## Discussion

Smoking and dyslipidemia are the traditional risk factors for CAD [19, 20]. In this study, Mediation model was used to examine whether lipid act as a mediator in the association between smoking and the risk of CAD. Mediation analyses showed that among the effects of smoking on CAD, 17.52% was mediated by lipid, among which HDL-C and TG accounted for 11.16 and 6.36%, respectively. Further analyses showed that the effect was also partially mediated by TG/HDL-C, which was accounted for 28%.

This research also verified that smoking was associated with the increased risk of CAD, which is consistent with previous studies [21]. In 1958, Hammond EC et al. found that the men who smoked 20 cigarettes per day were twice as likely to die of CAD compared with nonsmokers after tracking 187,783 people for 44 months [22]. In recent years, studies have also reported that smoking is associated with the development of CAD [23–25]. The “INTERHEART study” that was conducted on 29,972 samples also showed that smoking is responsible for the global acute myocardium infarction, and declared that smokers have a 1.95-fold increased risk of non-fatal myocardial infarction compared with Non-smokers [26].

In addition, lipid also plays a key role in the development of CAD. A series of studies has shown that TG, TC and LDL-C are positively associated with the risk of CAD, while HDL-C is inversely related to the risk of CAD [27, 28]. Furthermore, more and more attention has been paid to the predictive significance of TG/HDL-C on CAD in recent years [29]. A retrospective cohort study found TG/HDL-C to be associated with an increased cardiovascular mortality in female patients on peritoneal dialysis [30]. In our study, the abnormal levels of TG, TC, HDL-C and TG/HDL-C were associated with the CAD risk, but there was a negative correlation between TC and CAD risk, and no association was founded between LDL-C and CAD risk. This phenomenon might be caused by 59.28% patients in our study who had received statin therapy, which has a significant impact on TC and LDL-C levels so as to caused the underestimation on the lipid levels and CAD risk.

For a long time in the past, the mechanism by which smoking causes CAD was unclear. It was only recently that this mechanism has been generally understood, including oxidative stress, inflammation, lipid modification, vascular and endothelial dysfunction and others [31–35]. Traditional epidemiological researches found that lipid might be one of the mediators between smoking and the CAD occurrence. Previous studies showed that Nicotine found in tobacco induces a decrease in MAP kinase phosphatase-1 (MKP1), and leading to the activation of p38 mitogen-activated protein kinases and C-jun N-terminal kinases. These eventually promoting the loss of insulin-mediated lipid inhibition [32]. Therefore, nicotine increases lipolysis, which lead to changes in lipid levels and an increased risk of CAD, but it is still controversial [33]. Indeed, in our study, the abnormal levels of TG and HDL-C accounted for 6.36 and 11.16% effects on smoking and CAD risk respectively. And also, TG/HDL-C ratio largely mediated the relationship between smoking and CAD risk, which might be of great significance in predicting smoking played important roles in the pathogenesis of CAD.

## Study strengths and limitations

Although there were some strengths in this study, including the relatively large sample size and the mediating analyses to quantify the role of lipids in the association between smoking and CAD, some limitations also should be mentioned. Firstly, all the cases were selected from hospital, some of them might be not newly diagnosed by CAD, and they might had taken statins medicines in advance to decrease the lipid levels, which could be underestimated the effects of smoking on lipid levels. In addition, the drug information of the control group could’t be obtained due to the shortcomings in the questionnaire, so the study couldn’t put the medication information into statistical analysis. Secondly, all the CAD patients were diagnosed by coronary angiography, while the control group were selected from the community without the detection of coronary angiography, and based on the self-report and electrocardiograms examination to exclude the CAD status. This limitation might cause the selection bias of the research. Thirdly, no exact molecular mechanism had been explored in this study. The mediation analysis should be based on reasonable causal hypothesis for verification analysis, and in this original research, the lipid mediators had already been tested playing an important role in the CAD occurrence and progression. Finally, although some confounders have been adjusted in the regression analysis, there may still be some residual confounding factors that had not been adjusted, and this result should be verified by the further researches in multiple regions.

## Conclusion

In summary, the study found a significant association between smoking and CAD among chinese and the association was partially mediated by lipids. The study provides a clue on the mediation effect of lipids on the relationship between smoking and CAD risks, which is a novel insight to the progression of CAD. Further studies should be conducted to verify the findings and determine the elusive mechanism behind them.

## Data Availability

Not applicable.

## Abbreviations

CVDs: Cardiovascular diseases
CAD: Coronary artery disease
TC: Total Cholesterol
HDL-C: High-density lipoprotein-Cholesterol
LDL-C: Low-density lipoprotein-Cholesterol
TG: Triglyceride
TG/HDL-C: The ratio of TG to HDL-C
BMI: Body mass index
HBP: Hypertension
DM: Diabetes mellitus
WBC: White blood cells
